# Supplementary material for: Average semivariance yields accurate estimates of the fraction of marker-associated genetic variance and heritability in complex trait analyses
Source: PLoS Genet. 2021 Aug 26;17(8):e1009762. doi: 10.1371/journal.pgen.1009762 (PMC8425577; doi:10.1371/journal.pgen.1009762)
Supplement: S1 Table — Normally distributed phenotypic observations were simulated for 21 study designs and associated linear mixed models by varying the number of observations (n = nG × rG), the number of entries (nG), the number of replications/entry (rG), the number of marker loci (m), nM = 3 genotypes/marker locus, the number of entries/marker genotype (nG:M), and marker heritability (HM2). One thousand samples of size n were simulated for each study design. The segregation of a single marker locus in an F2 population was simulated in study design 4 The number of entries nested in marker genotypes for study design 4 was equivalent to the expected number for the segregation of a co-dominant DNA marker in a population segregating 1 AA : 2 Aa : 1 aa for a single marker locus. In this example, there are 135 entries nested in AA, 270 entries nested in Aa, and 135 entries nested in aa and each are replicated 5 times.simulates the segregation of a single locus in an F2 population The number of entries/genotype for study design 4. (PDF) [file pgen.1009762.s001.pdf]

| Design | $m$ | $n_{G:M}$ | $n_G$ | $r_G$ | $n$ | $H_M^2$ |
|---|---|---|---|---|---|---|
| 1 | 1 | 180 | 540 | 5 | 2,700 | (0.0, 1.0) |
| 2 | 2 | 180 | 540 | 5 | 2,700 | (0.0, 1.0) |
| 3 | 3 | 180 | 540 | 5 | 2,700 | (0.0, 1.0) |
| 4[a] | 1 | 135 : 270 : 135 | 540 | 5 | 2,700 | (0.0, 1.0) |
| 5[b] | 1 | (126, 180) | (486, 540) | (0, 5) | 2,430 | (0.0, 1.0) |
| 6[c] | 1 | (0, 180) | (360, 540) | (0, 5) | 1,800 | (0.0, 1.0) |
| 7 | 1 | 300 | 900 | 1 | 900 | 0.50 |
| 8 | 1 | 300 | 900 | 2 | 1,800 | 0.50 |
| 9 | 1 | 300 | 900 | 5 | 4,500 | 0.50 |
| 10 | 1 | 300 | 900 | 10 | 9,000 | 0.50 |
| 11 | 1 | 300 | 900 | 20 | 18,000 | 0.50 |
| 12 | 1 | 150 | 450 | 5 | 2,250 | 0.50 |
| 13 | 1 | 300 | 900 | 5 | 4,500 | 0.50 |
| 14 | 1 | 600 | 1,800 | 5 | 9,000 | 0.50 |
| 15 | 1 | 1200 | 3,600 | 5 | 18,000 | 0.50 |
| 16 | 1 | 2,400 | 7,200 | 5 | 36,000 | 0.50 |
| 17 | 1 | 150 | 450 | 5 | 2,250 | 0.05 |
| 18 | 1 | 150 | 450 | 5 | 2,250 | 0.25 |
| 19 | 1 | 150 | 450 | 5 | 2,250 | 0.50 |
| 20 | 1 | 150 | 450 | 5 | 2,250 | 0.75 |
| 21 | 1 | 150 | 450 | 5 | 2,250 | 0.95 |

[a]The number of entries nested in marker genotypes for study design 4 was equivalent to the expected number for the segregation of a co-dominant DNA marker in a population segregating 1 *AA* : 2 *Aa* : 1 aa for a single marker locus. In this example, there are 135 entries nested in *AA*, 270 entries nested in *Aa*, and 135 entries nested in *aa* and each are replicated 5 times.

[b]The numerical ranges for $r_M$, $n_G$, and $r_G$ varied among simulated samples for study design 5 because 10% of the phenotypic observations were randomly deleted to create unbalanced (missing) data. This design is equal to design 1, but with 10% of the data missing completely at random. We did not constrain how observations were removed and the to obtain values in range shown in columns $r_{G:M}$, $n_G$, and $r_G$.

[c]The numerical ranges for $r_M$, $n_G$, and $r_G$ varied among simulated samples for study design 6 because 33% of the phenotypic observations were randomly deleted to create unbalanced (missing) data. This design is equal to design 1, but with 33% of the data missing completely at random. We did not constrain how observations were removed and the to obtain values in range shown in columns $r_{G:M}$, $n_G$, and $r_G$.
