# Supplementary figures and images for: Average semivariance yields accurate estimates of the fraction of marker-associated genetic variance and heritability in complex trait analyses
Source: PLoS Genet. 2021 Aug 26;17(8):e1009762. doi: 10.1371/journal.pgen.1009762 (PMC8425577; doi:10.1371/journal.pgen.1009762)

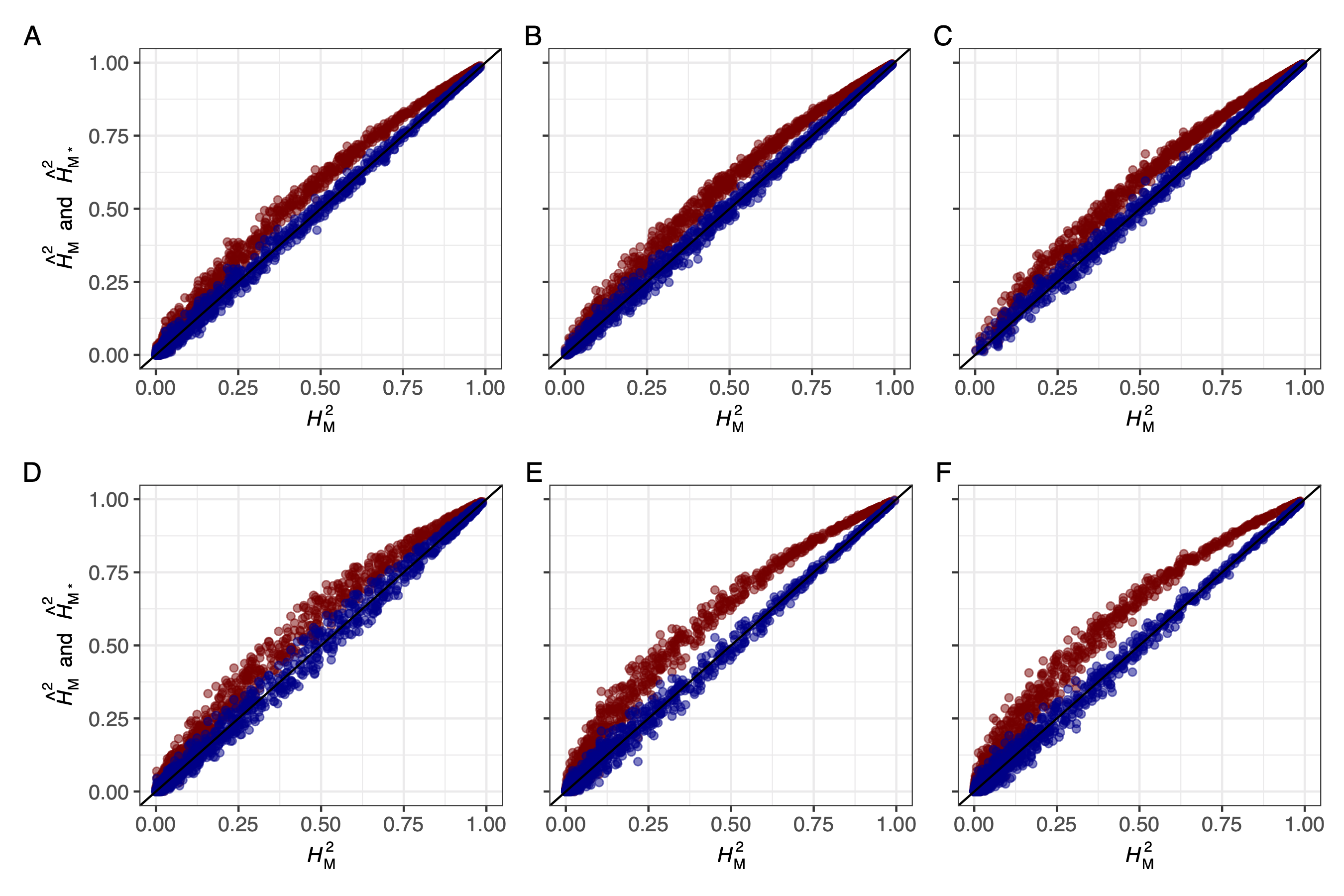

Supplement: S1 Fig — AMV and ASV estimates of HM2 when σG2 from LMM (1) is replaced with σ^M2+σ^G:M2 for AMV from LMM (2) or kMσ^M2+σ^G:M2 for ASV. Estimates are shown for 1,000 segregating populations simulated for different numbers of entries (nG individuals, families, or strains), five replications/entry (rG = 5), true marker heritability (HM2) ranging from 0 to 1, and one to three marker loci with three genotypes/marker locus (nM1 = 3). The AMV estimates (shown in red) equal σ^M2/(σ^M2+σ^G:M2+rG−1σ^ϵ2), whereas the ASV estimates (shown in blue) equal kMσ^M2/(kMσ^M2+σ^G:M2+rG−1σ^ϵ2). AMV estimates of marker heritability (H^M2; red highlighted observations) and ASV estimates of marker heritability (H^M*2; blue highlighted observations) are shown for: (A) one locus with balanced data for nG = 540 entries (study design 1); (B) two marker loci with interaction (M1, M2, and M1 × M2) and balanced data for nG = 540 (study design 2); (C) three marker loci with interactions (M1, M2, M3, M1 × M2, M1 × M3, M2 × M3, and M1 × M2 × M3) and balanced data for nG = 540 (study design 3); (D) a population segregating 1:2:1 for a single marker locus with rG:M = 135 entries for both homozygotes and rG:M = 270 heterozygous entries, and nG = 540 (study design 4); (E) one locus with 10% randomly missing data among 540 entries (study design 5); and (F) one locus with 33% randomly missing data among 540 entries (study design 6). Study design details are shown in S1 Table. (TIFF) [file pgen.1009762.s002.tiff]

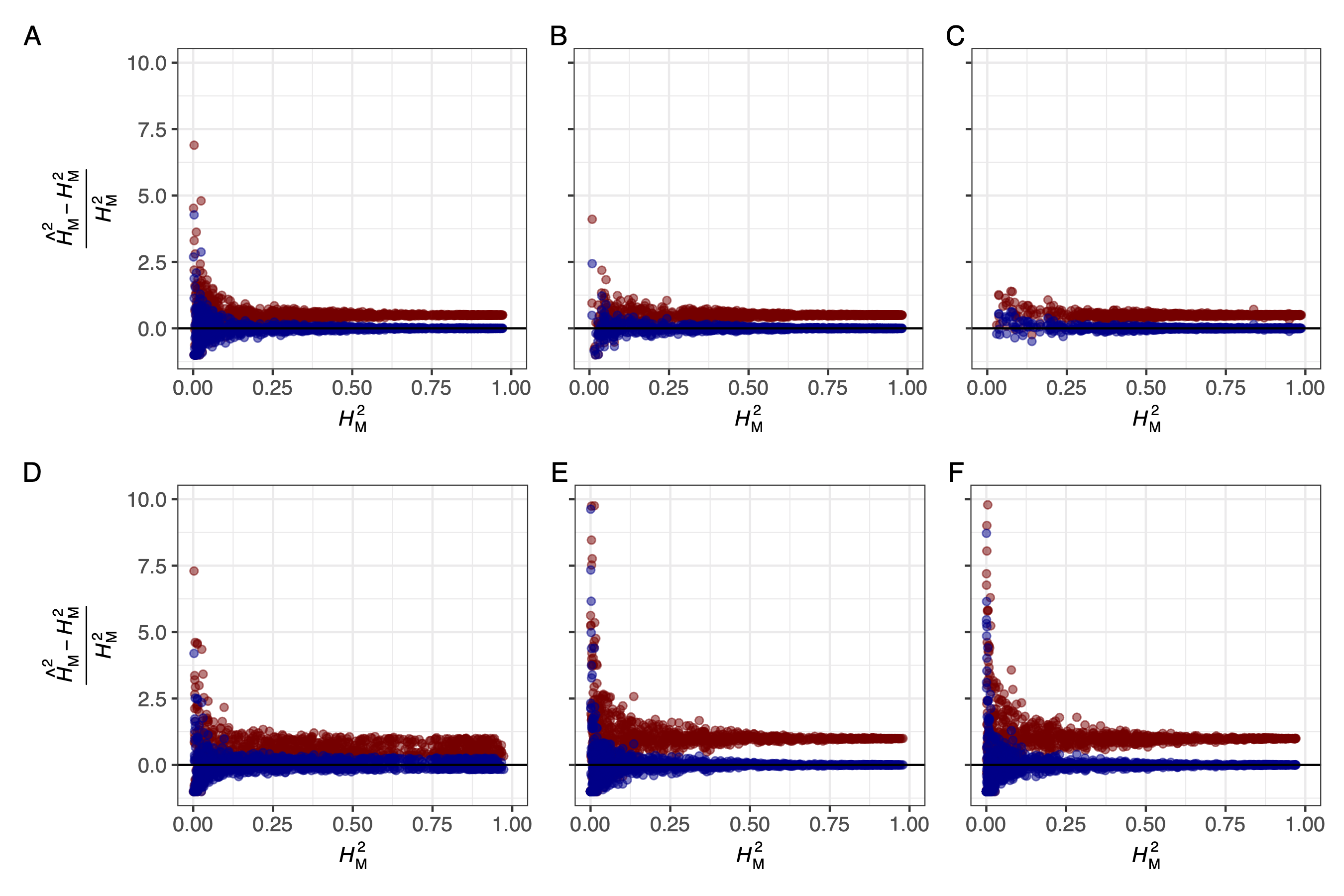

Supplement: S2 Fig — Relative biases of AMV and ASV estimates of HM2 are shown for 1,000 segregating populations simulated for different numbers of entries (nG individuals, families, or strains), five replications/entry (rG = 5), true marker heritability (HM2) ranging from 0 to 1, and one to three marker loci with three genotypes/marker locus (nM1 = 3). AMV estimates of marker heritability (H^M2; red highlighted observations) and ASV estimates of marker heritability (H^M*2; blue highlighted observations) are shown for: (A) one locus with balanced data for nG = 540 entries (study design 1); (B) two marker loci with interaction (M1, M2, and M1 × M2) and balanced data for nG = 540 (study design 2); (C) three marker loci with interactions (M1, M2, M3, M1 × M2, M1 × M3, M2 × M3, and M1 × M2 × M3) and balanced data for nG = 540 (study design 3); (D) an population segregating 1:2:1 for one marker locus with rG:M = 135 entries for both homozygotes and rG:M = 270 heterozygous entries, and nG = 540 (study design 4); (E) one locus with 10% randomly missing data among 540 entries (study design 5); and (F) one locus with 33% randomly missing data among 540 entries (study design 6). Study design details are shown in S1 Table. (TIFF) [file pgen.1009762.s003.tiff]
