# Supplementary material for: Average semivariance yields accurate estimates of the fraction of marker-associated genetic variance and heritability in complex trait analyses
Source: PLoS Genet. 2021 Aug 26;17(8):e1009762. doi: 10.1371/journal.pgen.1009762 (PMC8425577; doi:10.1371/journal.pgen.1009762)
Supplement: S1 Text — (PDF) [file pgen.1009762.s004.pdf]

S1 ASV Estimator of the Fraction of the Genetic Variance Associated With a Single Marker Locus for Unbalanced Data

We developed AMV and ASV estimators of p and H^2_M for analyses of balanced data because of their algebraic simplicity; however, phenotypic and genotypic data are nearly always unbalanced in practice. Here we extend the bias-correction solutions developed for balanced data to unbalanced data. ASV estimators of σ^2_M and $\sigma^2_{G:M}$ are developed here for a single marker locus (M) with unbalanced data. The phenotypic observations are entry-means ($\bar{y}_{hi\bullet}$).

The ASV estimator of the genetic variance associated with marker locus M for LMM (2) in its general form is:

$$\hat{\theta}^{ASV}_M = (n_G - 1)^{-1}\hat{\sigma}^2_M tr(Z_{u_M} Z^T_{u_M} D_{n_G}) = \frac{n_G - n_G^{-1}\sum_h n^2_{G:M_h}}{df_G}\hat{\sigma}^2_M = k_M\hat{\sigma}^2_M \qquad \text{(S1)}$$

where $\hat{\sigma}^2_M$ is the genetic variance associated with the marker locus effect, df_G is the degrees of freedom for entries, and $Z_{u_M} = \oplus_h 1_{n_{G:M_h}}$. $\hat{\theta}^{ASV}_M$ is a k_M-bias corrected estimate of the marker-associated genetic variance. This form of k_M can be thought of as the general form of the correction term relative to (12) which is specific to balanced study designs.

The ASV estimate of the residual genetic variance among entries nested in the marker locus M is:

$$\hat{\theta}^{ASV}_{G:M} = (n_G - 1)^{-1}\hat{\sigma}^2_{G:M} tr(Z_{u_{G:M}} Z^T_{u_{G:M}} D_{n_G}) = \frac{n_G - 1}{n_G - 1}\hat{\sigma}^2_{G:M} = \hat{\sigma}^2_{G:M} \qquad \text{(S2)}$$

where $\sigma^2_{G:M}$ is the residual genetic variance among entries nested in the marker locus effect and $Z_{u_{G:M}}$ is a n_G identity matrix.

From (S1), the ASV estimator of p for a single marker locus (M) with unbalanced data is:

$$\hat{p}_* = \frac{\hat{\theta}^{ASV}_M}{\hat{\theta}^{ASV}_G} = \frac{k_M\hat{\theta}^{AMV}_M}{\hat{\theta}^{AMV}_G} = \frac{k_M\hat{\sigma}^2_M}{\hat{\sigma}^2_G} = \frac{n_G - n_G^{-1}\sum_h n^2_{G:M_h}}{df_G} \times \frac{\hat{\sigma}^2_M}{\hat{\sigma}^2_G} \qquad \text{(S3)}$$

Hence, ASV yields unbiased estimates of the fraction of the genetic variance associated with the M locus (p) for unbalanced data. AMV estimates of p can be bias-corrected using the k_M coefficient shown above for unbalanced data. We review this here because most of the currently available linear mixed model software solutions produce AMV estimates, e.g., REML estimates of σ^2_M from LMM (2) are AMV estimates.
