# Supplementary material for: Average semivariance yields accurate estimates of the fraction of marker-associated genetic variance and heritability in complex trait analyses
Source: PLoS Genet. 2021 Aug 26;17(8):e1009762. doi: 10.1371/journal.pgen.1009762 (PMC8425577; doi:10.1371/journal.pgen.1009762)
Supplement: S2 Text — (PDF) [file pgen.1009762.s005.pdf]

S2 ASV Estimator of the Fraction of the Genetic Variance Associated with Two Marker Loci for Unbalanced Data

ASV estimators of σ^2_M and $\sigma^2_{G:M}$ are developed here for two marker loci ($M1$ and $M2$). The phenotypic observations are entry-means ($\bar{y}_{hij\bullet}$), the underlying data are unbalanced, and the LMM for the entry-mean analysis is:

$$\bar{y}_{hij\bullet} = \mu + M1_h + M2_i + M1 \times M2_{hi} + G : M_{hi(j)} + \bar{\epsilon}_{hij\bullet} \quad \text{(S4)}$$

where $\bar{y}_{hij\bullet}$ is the entry-mean, μ is the population mean, h = 1, 2, or 3, i = 1, 2, or 3, j = 1, 2, ..., n_G, k = 1, 2, ..., r_G, $M1_h$ is the random effect of marker locus 1 with $var(M1_h) = \sigma^2_{M1}$, $M2_i$ is the random effect of marker locus 2 with $var(M2_i) = \sigma^2_{M2}$, $(M1 \times M2)_{hi}$ is the random effect of the interaction between marker loci 1 and 2 with $var(M1 \times M2_{hi}) = \sigma^2_{M1\times M2}$, $G : M_{hi(j)}$ is the random effect of entries nested in marker loci with $var(G : M_{hi(j)}) = \sigma^2_{G:M}$, M refers to the $M1 \times M2$ interaction, and $\bar{\epsilon}_{hij\bullet}$ is the residual with $var(\bar{\epsilon}_{hij\bullet}) = r_G^{-1}\sigma^2_\epsilon$.

The ASV estimator of the genetic variance associated with marker locus $M1$ from LMM (S4) is:

$$\hat{\theta}^{ASV}_{M1} = (n_G - 1)^{-1}\hat{\sigma}^2_{M1} tr(Z_{u_{M1}} Z^T_{u_{M1}} D_{n_G}) = \frac{n_G - n_G^{-1}\sum_h n^2_{G:M1_h}}{df_G}\hat{\sigma}^2_{M1} = k_{M1}\hat{\sigma}^2_{M1} \quad \text{(S5)}$$

where $Z_{u_{M1}} = \oplus_h 1_{n_{G:M1_h}}$ as is the incidence matrix for $M1$, σ^2_{M1} is the genetic variance associated with marker locus $M1$, k_{M1} is the bias-correction factor for $M1$. Similarly, the ASV estimator of the genetic variance associated with marker locus $M2$ from LMM (S4) is:

$$\hat{\theta}^{ASV}_{M2} = (n_G - 1)^{-1}\hat{\sigma}^2_{M2} tr(Z_{u_{M2}} Z^T_{u_{M2}} D_{n_G}) = \frac{n_G - n_G^{-1}\sum_i n^2_{G:M2_i}}{df_G}\hat{\sigma}^2_{M2} = k_{M2}\hat{\sigma}^2_{M2} \quad \text{(S6)}$$

where $Z_{u_{M2}} = \oplus_i 1_{n_{G:M2_i}}$ is the incidence matrix for $M2$.

The ASV estimator of the genetic variance associated with the interaction between marker loci $M1$ and $M2$ is:

$$\begin{aligned}\hat{\theta}^{ASV}_{M1\times M2} &= (n_G - 1)^{-1}\hat{\sigma}^2_{M1\times M2} tr(Z_{u_{M1\times M2}} Z^T_{u_{M1\times M2}} D_{n_G}) \quad \text{(S7)}\\ &= \frac{n_G - n_G^{-1}\sum_{hi} n^2_{G:M_{hi}}}{df_G}\hat{\sigma}^2_{M1\times M2} = k_{M1\times M2}\hat{\sigma}^2_{M1\times M2}\end{aligned}$$

where $Z_{u_{M1\times M2}} = \oplus_{hi} 1_{n_{M1\times M2_{hi}}}$ is the incidence matrix for the $M1$ by $M2$ interaction, $I_{n_{M1\times M2}}$ is a $n_{M1\times M2}$ identity matrix, $1_{n_{G:M}}$ is a , $k_{M1\times M2}$ is the bias-correction factor for the interaction, $\hat{\sigma}^2_{M1\times M2}$ is the genetic variance associated with the interaction, and $\theta^{ASV}_{M1\times M2} \neq \sigma^2_{M1\times M2}$.

The ASV estimator of the variance associated with the residual genetic variance among entries nested in marker loci $M1$ and $M2$ is:

$$\hat{\theta}^{ASV}_{G:M} = (n_G - 1)^{-1}\hat{\sigma}^2_{G:M} tr(Z_{u_{G:M}} Z^T_{u_{G:M}} D_{n_G}) = \frac{n_G - 1}{n_G - 1}\hat{\sigma}^2_{G:M} = \hat{\sigma}^2_{G:M} \quad \text{(S8)}$$

where $Z_{u_{G:M}} = I_{n_G}$ is a n_G incidence matrix.

Hence, as for the two-marker analysis, ASV yields k_M-bias corrected estimates of the marker-associated genetic variance for two loci ($M1$ and $M2$) for unbalanced data:

$$\hat{p}_* = \frac{\hat{\theta}_{M1}^{ASV} + \hat{\theta}_{M2}^{ASV} + \hat{\theta}_{M1\times M2}^{ASV}}{\hat{\theta}_{G}^{ASV}} = \frac{k_{M1}\hat{\sigma}^2_{M1} + k_{M2}\hat{\sigma}^2_{M2} + k_{M1\times M2}\hat{\sigma}^2_{M1\times M2}}{\hat{\sigma}^2_G} \tag{S9}$$

The genetic variance associated with marker loci can be partitioned into intra-locus (additive and dominance) and inter-locus (additive $\times$ additive, additive $\times$ dominance, and dominance $\times$ dominance) components. The example shown here includes all components: two degrees of freedom each for $M1$ and $M2$ and four degrees of freedom for $M1 \times M2$.

From (S5) and (**??**), the coefficients for bias-correcting AMV estimates of σ^2_{M1} (the genetic variance explained by $M1$) and σ^2_{M2} (the genetic variance explained by $M2$) are:

$$k_{M1} = \frac{n_G - n_G^{-1}\sum_h n^2_{G:M1_h}}{df_G} \tag{S10}$$

$$k_{M2} = \frac{n_G - n_G^{-1}\sum_i n^2_{G:M2_i}}{df_G} \tag{S11}$$

Similarly, from (S7), the coefficient for bias-correcting AMV estimates of $\sigma^2_{M1\times M2}$ (the genetic variance explained by the interaction between $M1$ and $M2$) is:

$$k_{M1\times M2} = \frac{n_G - n_G^{-1}\sum_{hi} n^2_{G:M1\times M2_{hi}}}{df_G} \tag{S12}$$

These k_M-coefficients, (S10) and (S12), can be substituted in (S9) to obtain bias-corrected estimates of p.
