# Supplementary material for: Average semivariance yields accurate estimates of the fraction of marker-associated genetic variance and heritability in complex trait analyses
Source: PLoS Genet. 2021 Aug 26;17(8):e1009762. doi: 10.1371/journal.pgen.1009762 (PMC8425577; doi:10.1371/journal.pgen.1009762)
Supplement: S3 Text — (PDF) [file pgen.1009762.s006.pdf]

S3 ASV Estimator of the Fraction of the Genetic Variance Associated with Three Marker Loci for Unbalanced Data

ASV estimators of the genetic variance associated with intra-locus and inter-locus effects of three marker loci are developed here for unbalanced data. As before, the phenotypic observations are entry-means ($\bar{y}_{hijk\bullet}$) and the LMM for the entry-mean analysis is:

$$\begin{aligned}\bar{y}_{hijk\bullet} =& \mu + M1_h + M2_i + M2_j + M1 \times M2_{hi} + M1 \times M3_{hj} + M2 \times M3_{ij} \\ &+ M1 \times M2 \times M3_{hij} + G : M_{hij(k)} + \bar{\epsilon}_{hijk\bullet}\end{aligned} \tag{S13}$$

where $\bar{y}_{hijk\bullet}$ is the entry-mean, μ is the population mean, $h = 1$, 2, or 3, $i = 1$, 2, or 3, $j = 1, 2, ..., n_G$, $k = 1, 2, ..., r_G$, $M1_h$ is the random effect of marker locus 1 with $var(M1_h) = \sigma^2_{M1}$, $M2_i$ is the random effect of marker locus 2 with $var(M2_i) = \sigma^2_{M2}$, $M3_j$ is the random effect of marker locus 2 with $var(M3_j) = \sigma^2_{M3}$, $M1 \times M2_{hi}$ is the random effect of the interaction between marker loci 1 and 2 with $var(M1 \times M2_{hi}) = \sigma^2_{M1\times M2}$, $M1 \times M3_{hj}$ is the random effect of the interaction between marker loci 1 and 3 with $var(M1 \times M3_{hj}) = \sigma^2_{M1\times M3}$, $M2 \times M3_{ij}$ is the random effect of the interaction between marker loci 2 and 3 with $var(M2 \times M3_{ij}) = \sigma^2_{M2\times M3}$, $M1 \times M2 \times M3_{hij}$ is the random effect of the interaction between marker loci 1, 2, and 3 with $var(M1 \times M2 \times M3_{hij}) = \sigma^2_{M1\times M2\times M3}$, $G : M_{hij(k)}$ is the random effect of entries nested in marker loci with $var(G : M_{hij(k)}) = \sigma^2_{G:M)}$, M refers to the $M1 \times M2 \times M3$ interaction, and $\bar{\epsilon}_{hijk\bullet}$ is the residual with $var(\bar{\epsilon}_{hijk\bullet}) = r_G^{-1}\sigma^2_\epsilon$.

The ASV estimator of the genetic variance associated with marker locus $M1$ from LMM (S13) is:

$$\hat{\theta}^{ASV}_{M1} = (n_G - 1)^{-1}\hat{\sigma}^2_{M1} tr(Z_{u_{M1}} Z^T_{u_{M1}} D_{n_G}) = \frac{n_G - n_G^{-1}\sum_h n^2_{G:M1_h}}{df_G}\hat{\sigma}^2_{M1} \tag{S14}$$

where $P = I_{n_G} - n_G^{-1}J_{n_G}$, J_{n_G} is a n_G unit matrix, $Z_{u_{M1}} = \oplus_h 1_{n_{G:M1_h}}$ is the incidence matrix for $M1$. Substituting the appropriate values for marker loci $M2$ or $M3$ into (S14) yields the ASV estimators for these loci: $\hat{\theta}^{ASV}_{M2}$ and $\hat{\theta}^{ASV}_{M3}$.

The ASV estimator of the genetic variance associated with the two-locus interaction between marker loci $M1$ and $M2$ is:

$$\begin{aligned}\hat{\theta}^{ASV}_{M1\times M2} &= (n_G - 1)^{-1}\hat{\sigma}^2_{M1\times M2} tr(Z_{u_{M1\times M2}} Z^T_{u_{M1\times M2}} D_{n_G}) \\ &= \frac{n_G - n_G^{-1}\sum_{hi} n^2_{G:M1\times M2_{hi}}}{df_G}\hat{\sigma}^2_{M1\times M2}\end{aligned} \tag{S15}$$

where $Z_{u_{M1:M2}} = \oplus_{hi} 1_{n_{M1:M2_{hi}}}$ is the incidence matrix for $M1 \times M2$ interaction. ASV estimates of the other two-locus interactions are similarly defined. The ASV estimator of the genetic variance associated with the three-locus interaction ($M1 \times M2 \times M3$) is:

$$\begin{aligned}\hat{\theta}^{ASV}_{M1\times M2\times M3} &= (n_G - 1)^{-1}\hat{\sigma}^2_{M1\times M2\times M3} tr(Z_{u_{M1\times M2\times M3}} Z^T_{u_{M1\times M2\times M3}} D_{n_G}) \\ &= \frac{n_G - n_G^{-1}\sum_{hij} n^2_{G:M1\times M2\times M3_{hij}}}{df_G}\hat{\sigma}^2_{M1\times M2\times M3}\end{aligned} \tag{S16}$$

where $Z_{u_{M1\times M2\times M3}} = \oplus_{hij} 1_{n_{M1\times M2\times M3_{hij}}}$ is the incidence matrix for the three-locus interaction. Finally, the ASV estimator of the residual genetic variance among entries nested in marker loci is:

$$\hat{\theta}^{ASV}_{G:M} = (n_G - 1)^{-1}\hat{\sigma}^2_{G:M} tr(Z_{u_{G:M}} Z^T_{u_{G:M}} D_{n_G}) = \frac{n_G - 1}{n_G - 1}\hat{\sigma}^2_{G:M} = \hat{\sigma}^2_{G:M} \tag{S17}$$

where $Z_{u_{G:M}} = I_{n_G}$ is a n_G identity matrix.

Hence, as for the three-marker analysis, ASV yields k_M-bias corrected estimates of the marker-associated genetic variance for three loci ($M1$, $M2$, and $M3$) for unbalanced data:

$$\begin{aligned} \hat{p}_* &= \frac{\sum_{i=1}^{3} \hat{\theta}^{ASV}_{Mi} + \sum_{j=1}^{2}\sum_{k=j+1}^{3} \hat{\theta}^{ASV}_{Mj\times Mk} + \hat{\theta}^{ASV}_{M1\times M2\times M3}}{\hat{\theta}^{ASV}_{G}} \\ &= \frac{\sum_{i=1}^{3} k_{Mi}\hat{\sigma}^2_{Mi} + \sum_{j=1}^{2}\sum_{k=j+1}^{3} k_{Mj\times Mk}\hat{\sigma}^2_{Mj\times Mk} + k_{M1\times M2\times M2}\hat{\sigma}^2_{M1\times M2\times M3}}{\hat{\sigma}^2_G} \end{aligned} \tag{S18}$$

where $i = 1, 2, 3$, $j = 1, 2$, and $k = 2, 3$ and $j \neq k$ are used to specify $M1$, $M2$, $M3$ and all two-way locus-locus interactions.

From (S14), the k_M-coefficient for bias-correcting AMV estimates of σ^2_{M1}, σ^2_{M1}, and σ^2_{M1} are:

$$k_{M1} = \frac{n_G - n_G^{-1}\sum_h n^2_{G:M1_h}}{df_G} \tag{S19}$$

$$k_{M2} = \frac{n_G - n_G^{-1}\sum_i n^2_{G:M2_i}}{df_G} \tag{S20}$$

$$k_{M3} = \frac{n_G - n_G^{-1}\sum_j n^2_{G:M3_j}}{df_G} \tag{S21}$$

Similarly, from (S15), the k_M-coefficient for bias-correcting AMV estimates of $\sigma^2_{M1\times M2}$, $\sigma^2_{M1\times M2}$, and $\sigma^2_{M2\times M3}$ are:

$$k_{M1\times M2} = \frac{n_G - n_G^{-1}\sum_{hi} n^2_{G:M1\times M2_{hi}}}{df_G} \tag{S22}$$

$$k_{M1\times M3} = \frac{n_G - n_G^{-1}\sum_{hj} n^2_{G:M1\times M3_{hj}}}{df_G} \tag{S23}$$

$$k_{M2\times M3} = \frac{n_G - n_G^{-1}\sum_{ij} n^2_{G:M2\times M3_{ij}}}{df_G} \tag{S24}$$

From (S16), the k_M-coefficient for bias-correcting AMV estimates of $\sigma^2_{M1\times M2\times M3}$ (the genetic variance explained by the three-way marker loci interaction) is:

$$k_{M1\times M2\times M3} = \frac{n_G - n_G^{-1}\sum_{hij} n^2_{G:M_{hij}}}{df_G} \tag{S25}$$

These k_M-coefficients, (S19) and (S25), are substituted in (S18) to obtain bias-corrected REML estimates of p.
