# Supplementary material for: Average semivariance yields accurate estimates of the fraction of marker-associated genetic variance and heritability in complex trait analyses
Source: PLoS Genet. 2021 Aug 26;17(8):e1009762. doi: 10.1371/journal.pgen.1009762 (PMC8425577; doi:10.1371/journal.pgen.1009762)
Supplement: S4 Text — (PDF) [file pgen.1009762.s007.pdf]

S4 Biases of AMV and ASV Estimators of Marker-Associated Variance

The true value of the variance for the between-entry effect in LMM (1) is:

$$s_G^2 = (n_G - 1)^{-1} \sum_{j=1}^{n_G} (g_j - \bar{g}_{\bullet})^2 \tag{S26}$$

where $\bar{g}$ are the entry means, $\bar{g}_j$ is the jth element of $\bar{g}$, $j = 1, 2, 3, ..., n_G$, and $\bar{g}_{\bullet}$ is the mean of $\bar{g}$. Note that $E(s_G^2) = E(\hat{\sigma}_G^2) = \sigma_G^2$. The variance of the residual effect in LMM (1) and (2) is:

$$s_{\bar{\epsilon}}^2 = (n_G - 1)^{-1} \sum_{j=1}^{n_G} (\bar{\epsilon}_j - \bar{\epsilon}_{\bullet})^2 \tag{S27}$$

where $\bar{\epsilon}$ are the mean residuals across entries, $\bar{\epsilon}_j$ is the jth element of $\bar{\epsilon}$, $j = 1, 2, 3, ..., n_G$, and $\bar{\epsilon}_{\bullet}$ is the mean of $\bar{\epsilon}$. We note that $E(s_{\bar{\epsilon}}^2) = E(\hat{\sigma}_{\bar{\epsilon}}^2) = \sigma_{\bar{\epsilon}}^2$. The variance for the entries nested in M effect in LMM (2) is:

$$s_{G:M}^2 = (n_G - 1)^{-1} \sum_{j=1}^{n_G} (\bar{q}_j - \bar{q}_{\bullet})^2 \tag{S28}$$

where $\bar{q}_j$ is the effect of the jth entry nested in marker locus 1, $j = 1, 2, 3, ..., n_G$, and $\bar{q}_{\bullet}$ is the mean of $\bar{q}$ across entries. Note that $E(s_{G:M}^2) = E(\hat{\sigma}_{G:M}^2) = \sigma_{G:M}^2$. Finally, the variance for the effect of a single marker locus (M) in LMM (2) is:

$$s_M^2 = (n_G - 1)^{-1} \sum_{j=1}^{n_G} (m_j - \bar{m}_{\bullet})^2 \tag{S29}$$

where m_j is the effect of the jth genotype for a single marker locus, $j = 1, 2, 3, ..., n_G$, and $\bar{m}_{\bullet}$ is the mean of m across entries. Note that the summation in (S29) is over entries and not marker alleles. We demonstrate in the main body of this text that $E(s_M^2) \leq E(\hat{\sigma}_M^2) = \sigma_M^2$ which causes p and H_M^2 to be systematically overestimated. The expected value of s_M^2 is:

$$E(s_M^2) = (n_G - 1)^{-1} \sigma_M^2 tr(Z_{n_G} Z_{n_G}^T D_{n_G}) = \theta_M^{ASV} \tag{S30}$$

Note that the ASV estimator θ_M^{ASV} defines the expected value of s_M^2 which, as we demonstrate in the main text, is a fraction of the AMV estimator $\theta_M^{AMV} = E(\sigma_M^2)$. Our bias argument, supported by overwhelming simulation evidence, applies both in relation to the samples s_M^2 and $E(s_M^2)$, but we focus on the former as it is more tangible.

The bias of the AMV estimator of the marker-associated genetic variance is:

$$bias(\hat{\theta}_M^{AMV}) = E[\hat{\theta}_M^{AMV}] - s_M^2 \tag{S31}$$

where $\hat{\theta}_M^{AMV}$ is the AMV estimate of the marker associated genetic variance and $E[\hat{\theta}_M^{AMV}]$ is the expected value of $\hat{\theta}_M^{AMV}$. Similarly, the bias of the ASV estimator is:

$$bias(\hat{\theta}_M^{ASV}) = E[\hat{\theta}_M^{ASV}] - s_M^2 \tag{S32}$$

where $\hat{\theta}_M^{ASV}$ is the ASV estimator of the marker associated genetic variance and $E[\hat{\theta}_M^{ASV}]$ is the expected value of $\hat{\theta}_M^{ASV}$.

The relative bias of the AMV estimator is:

$$RB(\hat{\theta}_M^{AMV}) = \frac{E[\hat{\theta}_M^{AMV}] - s_M^2}{s_M^2} \tag{S33}$$

and the relative bias of the ASV estimator is:

$$RB(\hat{\theta}_M^{ASV}) = \frac{E[\hat{\theta}_M^{ASV}] - s_M^2}{s_M^2} \tag{S34}$$

The biases and relative biases were empirically estimated through computer simulations.
