# Supplementary material for: Average semivariance yields accurate estimates of the fraction of marker-associated genetic variance and heritability in complex trait analyses
Source: PLoS Genet. 2021 Aug 26;17(8):e1009762. doi: 10.1371/journal.pgen.1009762 (PMC8425577; doi:10.1371/journal.pgen.1009762)
Supplement: S5 Text — (PDF) [file pgen.1009762.s008.pdf]

S5 Sample Variances for AMV and ASV Estimators of p and H_M^2

Sample variances for AMV and ASV estimators of p and H_M^2 were developed using the delta method and basic covariance algebra. The sample variance of the AMV estimator of p is:

$$var(\hat{p}) \approx \left(\frac{\hat{\sigma}_M^2}{\hat{\sigma}_G^2}\right)^2 \left(\frac{var(\hat{\sigma}_M^2)}{(\hat{\sigma}_M^2)^2} + \frac{var(\hat{\sigma}_G^2)}{(\hat{\sigma}_G^2)^2} - \frac{2cov(\hat{\sigma}_M^2, \hat{\sigma}_G^2)}{\hat{\sigma}_M^2 \times \hat{\sigma}_G^2}\right) \tag{S35}$$

where $var(\hat{\sigma}_G^2)$ is the variance of σ_G^2, $var(\hat{\sigma}_M^2)$ is the variance of $\hat{\sigma}_M^2$, and $cov(\hat{\sigma}_M^2, \hat{\sigma}_G^2)$ is the covariance between $\hat{\sigma}_M^2$ and $\hat{\sigma}_G^2$. Similarly, the sample variance of the AMV estimator of H_M^2 is:

$$var(\hat{H}_M^2) \approx \left(\frac{\hat{\sigma}_M^2}{\hat{\sigma}_{\bar{P}}^2}\right)^2 \left(\frac{var(\hat{\sigma}_M^2)}{(\hat{\sigma}_M^2)^2} + \frac{var(\hat{\sigma}_{\bar{P}}^2)}{(\hat{\sigma}_{\bar{P}}^2)^2} - \frac{2cov(\hat{\sigma}_M^2, \hat{\sigma}_{\bar{P}}^2)}{\hat{\sigma}_M^2 \times \hat{\sigma}_{\bar{P}}^2}\right) \tag{S36}$$

where $cov(\hat{\sigma}_M^2, \hat{\sigma}_G^2)$ is the covariance between $\hat{\sigma}_M^2$ and $\hat{\sigma}_G^2$, $var(\hat{\sigma}_{\bar{P}}^2)$ is the variance for the phenotypic variance on an entry-mean basis ($\hat{\sigma}_{\bar{P}}^2$), and $cov(\hat{\sigma}_M^2, \hat{\sigma}_{\bar{P}}^2)$ is the covariance between $\hat{\sigma}_M^2$ and $\hat{\sigma}_{\bar{P}}^2$ from equation (7). The variance of $\hat{\sigma}_{\bar{P}}^2$ is:

$$var(\hat{\sigma}_{\bar{P}}^2) = var(\hat{\sigma}_G^2 + \hat{\sigma}_\epsilon^2/r_G) = var(\hat{\sigma}_G^2) + \frac{1}{r_G^2} var(\hat{\sigma}_\epsilon^2) + \frac{2}{r_G} cov(\hat{\sigma}_G^2, \hat{\sigma}_\epsilon^2) \tag{S37}$$

where $cov(\hat{\sigma}_M^2, \hat{\sigma}_\epsilon^2)$ is the covariance between $\hat{\sigma}_M^2$ and $\hat{\sigma}_\epsilon^2$. The covariance between $\hat{\sigma}_M^2$ and $\hat{\sigma}_G^2$) is:

$$cov(\hat{\sigma}_M^2, \hat{\sigma}_G^2) = cov(\hat{\sigma}_M^2, \hat{\sigma}_G^2) = cov(\hat{\sigma}_M^2, \hat{\sigma}_M^2 + \hat{\sigma}_{G:M}^2) = var(\hat{\sigma}_M^2) + cov(\hat{\sigma}_M^2, \hat{\sigma}_{G:M}^2) \tag{S38}$$

where $cov(\hat{\sigma}_M^2, \hat{\sigma}_{G:M}^2)$ is the covariance between $\hat{\sigma}_M^2$ and $\hat{\sigma}_{G:M}^2$. The covariance between $\hat{\sigma}_M^2$ and $\hat{\sigma}_{\bar{P}}^2$ from equation (7) is:

$$cov(\hat{\sigma}_M^2, \hat{\sigma}_{\bar{P}}^2) = cov(\hat{\sigma}_M^2, \hat{\sigma}_G^2 + \hat{\sigma}_\epsilon^2/r_G) = cov(\hat{\sigma}_M^2, \hat{\sigma}_G^2) + \frac{1}{r_G} cov(\hat{\sigma}_M^2, \hat{\sigma}_\epsilon^2) \tag{S39}$$

These sample variances can be extracted from the asymptotic variance-covariance matrices estimated with widely used software for linear mixed model analyses.

Using the delta method and recalling from equation (8) that $k_M\hat{\sigma}_M^2 + \hat{\sigma}_{G:M}^2 = \hat{\sigma}_G^2$, the sample variance of the ASV estimates of p is:

$$var(\hat{p}_{M*}) \approx \left(\frac{k_M\hat{\sigma}_M^2}{\hat{\sigma}_G^2}\right)^2 \left(\frac{var(\hat{\sigma}_M^2)}{(\hat{\sigma}_M^2)^2} + \frac{var(\hat{\sigma}_G^2)}{(\hat{\sigma}_G^2)^2} - \frac{2cov(\hat{\sigma}_M^2, \hat{\sigma}_G^2)}{\hat{\sigma}_M^2 \times \hat{\sigma}_G^2}\right) \tag{S40}$$

where $var(\hat{\sigma}_G^2)$ is the variance of σ_G^2, $var(k_M\hat{\sigma}_M^2)$ is the variance of $k_M\hat{\sigma}_M^2$, and $cov(k_M hat\sigma_M^2, \hat{\sigma}_G^2)$ is the covariance between $k_M\hat{\sigma}_M^2$ and $\hat{\sigma}_G^2$.

Similarly, the sample variance for the ASV estimator of H_M^2 is:

$$var(\hat{H}_{M*}^2) \approx \left(\frac{k_M\hat{\sigma}_M^2}{\hat{\sigma}_{\bar{P}}^2}\right)^2 \left(\frac{var(\hat{\sigma}_M^2)}{(\hat{\sigma}_M^2)^2} + \frac{var(\hat{\sigma}_{\bar{P}}^2)}{(\hat{\sigma}_{\bar{P}}^2)^2} - \frac{2cov(\hat{\sigma}_M^2, \hat{\sigma}_{\bar{P}}^2)}{\hat{\sigma}_M^2 \times \hat{\sigma}_{\bar{P}}^2}\right) \tag{S41}$$

where $cov(k_M\hat{\sigma}_M^2, \hat{\sigma}_G^2)$ is the covariance between $k_M\hat{\sigma}_M^2$ and $\hat{\sigma}_G^2$, $var(\hat{\sigma}_{\bar{P}}^2)$ is the variance for the phenotypic variance on an entry-mean basis, and $cov(k_M\hat{\sigma}_M^2, \hat{\sigma}_{\bar{P}}^2)$ is the

covariance between $k_M\hat{\sigma}^2_M$ and $\hat{\sigma}^2_{\bar{P}}$ from equation (7). The covariance between $k_M\hat{\sigma}^2_M$ and $\hat{\sigma}^2_{\bar{P}}$ from equation (2) is:

$$\begin{aligned} cov(k_M\hat{\sigma}^2_M, \hat{\sigma}^2_{\bar{P}}) &= cov(k_M\hat{\sigma}^2_M, \hat{\sigma}^2_G + \hat{\sigma}^2_\epsilon/r_G) \\ &= k_M cov(\hat{\sigma}^2_M, \hat{\sigma}^2_G) + \frac{k_M}{r_G} cov(\hat{\sigma}^2_M, \hat{\sigma}^2_\epsilon) \\ &= k^2_M var(\hat{\sigma}^2_M) + k_M cov(\hat{\sigma}^2_M, \hat{\sigma}^2_{G:M}) + \frac{k_M}{r_G} cov(\hat{\sigma}^2_M, \hat{\sigma}^2_\epsilon) \end{aligned} \tag{S42}$$

where $cov(\hat{\sigma}^2_M, \hat{\sigma}^2_{G:M})$ is the covariance between $\hat{\sigma}^2_M$ and $\hat{\sigma}^2_{G:M}$ from equation (2). The covariance between $k_M\hat{\sigma}^2_M$ and $\hat{\sigma}^2_G$ is:

$$\begin{aligned} cov(k_M\hat{\sigma}^2_M, \hat{\sigma}^2_G) &= k_M cov(\hat{\sigma}^2_M, \hat{\sigma}^2_G) \\ &= k_M cov(\hat{\sigma}^2_M, k_M\hat{\sigma}^2_M + \hat{\sigma}^2_{G:M}) \\ &= k^2_M var(\hat{\sigma}^2_M) + k_M cov(\hat{\sigma}^2_M, \hat{\sigma}^2_{G:M}) \end{aligned} \tag{S43}$$

We found that the sample variances of the ASV estimators are consistently smaller than AMV estimators of p and H^2_M by a factor of $1 - k_M$, as shown here:

$$\begin{aligned} \frac{var(\hat{p}_*)}{var(\hat{p})} &\approx \frac{\left(\frac{k_M\hat{\sigma}^2_M}{\hat{\sigma}^2_G}\right)^2 \left(\frac{var(\hat{\sigma}^2_M)}{(\hat{\sigma}^2_M)^2} + \frac{var(\hat{\sigma}^2_G)}{(\hat{\sigma}^2_G)^2} - \frac{2cov(\hat{\sigma}^2_M, \hat{\sigma}^2_G)}{\hat{\sigma}^2_M \times \hat{\sigma}^2_G}\right)}{\left(\frac{\hat{\sigma}^2_M}{\hat{\sigma}^2_G}\right)^2 \left(\frac{var(\hat{\sigma}^2_M)}{(\hat{\sigma}^2_M)^2} + \frac{var(\hat{\sigma}^2_G)}{(\hat{\sigma}^2_G)^2} - \frac{2cov(\hat{\sigma}^2_M, \hat{\sigma}^2_G)}{\hat{\sigma}^2_M \times \hat{\sigma}^2_G}\right)} \\ &= \frac{\left(\frac{k_M\hat{\sigma}^2_M}{\hat{\sigma}^2_G}\right)^2}{\left(\frac{\hat{\sigma}^2_M}{\hat{\sigma}^2_G}\right)^2} = \frac{k^2_M\left(\frac{\hat{\sigma}^2_M}{\hat{\sigma}^2_G}\right)^2}{\left(\frac{\hat{\sigma}^2_M}{\hat{\sigma}^2_G}\right)^2} = \frac{k^2_M}{1} < 1 \end{aligned} \tag{S44}$$

and

$$\begin{aligned} \frac{var(\hat{H}^2_{M*})}{var(\hat{H}^2_M)} &\approx \frac{\left(\frac{k_M\hat{\sigma}^2_M}{\hat{\sigma}^2_{\bar{P}}}\right)^2 \left(\frac{var(\hat{\sigma}^2_M)}{(\hat{\sigma}^2_M)^2} + \frac{var(\hat{\sigma}^2_{\bar{P}})}{(\hat{\sigma}^2_{\bar{P}})^2} - \frac{2cov(\hat{\sigma}^2_M, \hat{\sigma}^2_{\bar{P}})}{\hat{\sigma}^2_M \times \hat{\sigma}^2_{\bar{P}}}\right)}{\left(\frac{\hat{\sigma}^2_M}{\hat{\sigma}^2_{\bar{P}}}\right)^2 \left(\frac{var(\hat{\sigma}^2_M)}{(\hat{\sigma}^2_M)^2} + \frac{var(\hat{\sigma}^2_{\bar{P}})}{(\hat{\sigma}^2_{\bar{P}})^2} - \frac{2cov(\hat{\sigma}^2_M, \hat{\sigma}^2_{\bar{P}})}{\hat{\sigma}^2_M \times \hat{\sigma}^2_{\bar{P}}}\right)} \\ &= \frac{\left(\frac{k_M\hat{\sigma}^2_M}{\hat{\sigma}^2_{\bar{P}}}\right)^2}{\left(\frac{\hat{\sigma}^2_M}{\hat{\sigma}^2_{\bar{P}}}\right)^2} = \frac{k^2_M\left(\frac{\hat{\sigma}^2_M}{\hat{\sigma}^2_{\bar{P}}}\right)^2}{\left(\frac{\hat{\sigma}^2_M}{\hat{\sigma}^2_{\bar{P}}}\right)^2} = \frac{k^2_M}{1} < 1 \end{aligned} \tag{S45}$$

Importantly, k^2_M is the square of a value less than 1 indicating that the sampling variance of ASV estimates of p_* and H^2_{M*} are less than those of p and H^2_M.
